# Intravascular polarization-sensitive optical coherence tomography based on polarization mode delay

**DOI:** 10.1038/s41598-022-10709-8

**Published:** 2022-04-27

**Authors:** Yan Li, Sucbei Moon, Yuchen Jiang, Saijun Qiu, Zhongping Chen

**Affiliations:** 1 Beckman Laser Institute, University of California, Irvine, Irvine, CA 92617 USA; 2 Department of Physics, Kookmin University, Seoul, 02707 South Korea; 3 Department of Biomedical Engineering, University of California, Irvine, Irvine, CA 92697 USA; 4 The Cardiovascular Innovation and Research Center, University of California, Irvine, , Irvine, CA 92617 USA

**Keywords:** Atherosclerosis, Optical techniques

## Abstract

Intravascular polarization-sensitive optical coherence tomography (IV-PSOCT) provides depth-resolved tissue birefringence which can be used to evaluate the mechanical stability of a plaque. In our previous study, we reported a new strategy to construct polarization-sensitive optical coherence tomography in a microscope platform. Here, we demonstrated that this technology can be implemented in an endoscope platform, which has many clinical applications. A conventional intravascular OCT system can be modified for IV-PSOCT by introducing a 12-m polarization-maintaining fiber-based imaging probe. Its two polarization modes separately produce OCT images of polarization detection channels spatially distinguished by an image separation of 2.7 mm. We experimentally validated our IV-PSOCT with chicken tendon, chicken breast, and coronary artery as the image samples. We found that the birefringent properties can be successfully visualized by our IV-PSOCT.

## Introduction

Atherosclerosis is a progressive disease in which plaque buildup (i.e., fatty deposits) occurs within the lining of an artery^1,2^. Ruptured plaques cause 86% of heart attacks and 45% of brain aneurysms^3–5^. These high-risk, vulnerable plaques often remain clinically silent, and its initial manifestation can be sudden and lethal^6^. Detection methods that aim for early recognition of vulnerable plaques are, first and foremost, of critical importance for preventing deadly consequences^3^. Studies have demonstrated the association between plaque vulnerability and with its morphology, chemical composition, and biomechanical properties^7–15^: (1) Morphologically, the thickness of a fibrous cap is a reliable indicator of plaque vulnerability^9,16^; (2) Chemically, the intra-lesion lipid density and the cholesterol content have shown to be correlated with the vulnerability^9,17,18^, and; (3) Mechanically, collagen, which is secreted by smooth muscle cells that contain myosin and actin, is the extracellular matrix protein that imparts mechanical stability to a plaque^19^.

A variety of optical and non-optical imaging schemes have been developed for early diagnosis of vulnerable plaque. Intravascular ultrasound (IVUS) and intravascular optical coherence tomography (IVOCT) are commonly used in clinical practice to visualize layered structure of the vascular tissue. The large penetration depth of IVUS enables full-depth visualization of the coronary lumen, blood vessel wall, and atherosclerotic plaque formation. Benefiting from its micron-scale resolution, IVOCT has the capability to measure fibrous cap thickness^20,21^. To find molecular composition of the plaque, optical schemes such as intravascular near-infrared fluorescence and spectroscopy (NIRF and NIRS) have been considered which are capable of characterizing the intra-lesion lipid contents, but lack of depth resolvability^22–27^. Intravascular photoacoustic imaging can provide excellent molecular contrast in depth-resolved images while maintaining the superior imaging depth of ultrasound-based imaging^28–32^. However, none of these can directly provide the mechanical properties of fibrous cap which are very important information to assess the vulnerability. Biomechanically, shifting in local tissue elasticity is one of the key indicators as the stress in a fibrous cap is altered by its thickness and macrophage infiltration; more importantly, tissue elasticity can be used for identifying plaque type based on the composition-dependent biomechanical property of the plaque^12–15^.

Polarization-sensitive optical coherence tomography (PSOCT) is capable of providing depth-resolved sample birefringence^33,34^. Various techniques have been developed to construct PSOCT in a microscope platform. However, implement of PSOCT in an endoscope platform remains challenging. In the recent studies^19,35–37^, an advanced endoscopic scheme of IVOCT was proposed based on the PSOCT, where the polarization state was actively modulated while utilizing a specialized photodetector which can sense the polarization state of the sample light field. They found that the mechanical integrity and vulnerability of atherosclerotic plaques can be successfully evaluated from the birefringence information, which represents a significant step towards comprehensive diagnosis of atherosclerosis. However, a complicated algorithm is needed for reconstruction of tissue birefringence, which also degrades system’s axial resolution. For clinical viability of the technique, further development of an economic and simple approach of PSOCT imaging integrated with a miniaturized endoscopic catheter is required. In 2019, our group demonstrated a very simple method to construct PSOCT which utilizes a section of polarization-maintain fiber (PMF) for polarization detection channels without needs of active polarization modulation and complicated computation while maintaining original resolution^38^.

In this study, we further extend this study to propose an intravascular PSOCT (IV-PSOCT) which can acquire spatial distribution of the sample’s birefringence through a small intravascular imaging probe. We took advantage of polarization-maintaining fiber (PMF) which differentiates the polarization-channeled image information. A conventional OCT system can be modified for IV-PSOCT by introducing a 12-m PMF based imaging probe with ease. Ex vivo experiments were performed to demonstrate the performance of our IV-PSOCT.

## Results

### Microscopic PSOCT imaging test

**Figure 1:**
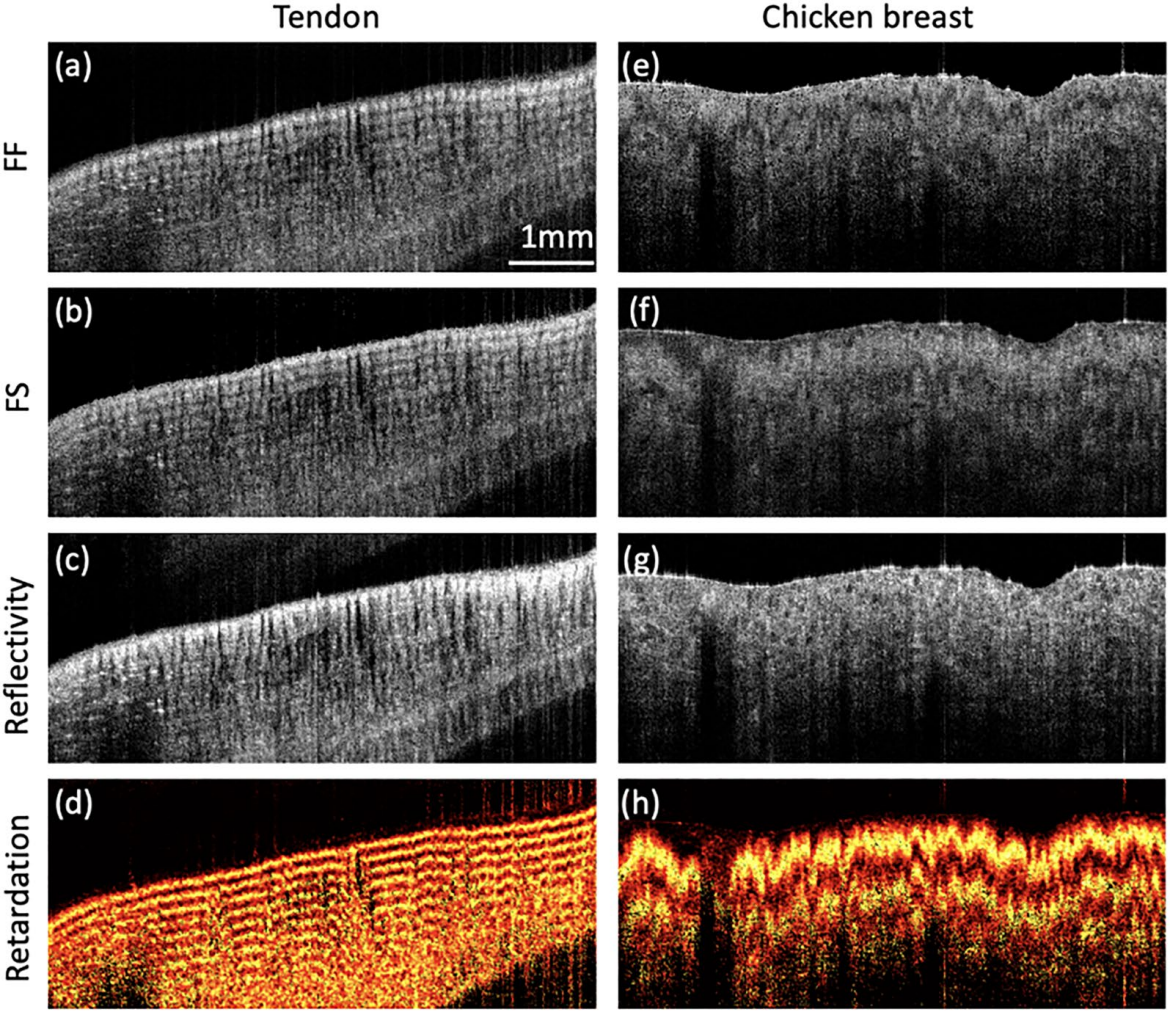
Images of chicken tendon and chicken breast. (**a**) FF image, (**b**) FS image, (**c**) the normal image of reflectivity, and (**d**) the map of sample-field retardation of chicken tendon. (**e**) FF image, (**f**) FS image, (**g**) the normal image of reflectivity, and (**h**) the map of sample-field retardation of chicken breast. Scale bar: 1 mm. The figure is generated by using Matlab R2021a (Version: 9.10.0.1602886, https://www.mathworks.com/products/new_products/release2021a.html).

Imaging capability of proposed method was first tested with a tissue sample of chicken tendon and chicken breast ex vivo using a microscopic PSOCT, as shown in Fig. 1. Figure 1a–d are fast–fast (FF) image and fast–slow (FS) image, reflectivity (normal OCT image), and sample-field retardation (tissue birefringence) of chicken tendon, respectively. Figure 1e–h are FF (fast-fast) image, FS (fast-slow) image, reflectivity, and sample-field retardation of chicken breast, respectively. The effect of the sample birefringence is clearly visualized in the pseudo-color map in Fig. 1d,h. Furthermore, tendon and chicken breast exhibit different levels of birefringence.

### Experiments with IV-PSOCT

**Figure 2:**
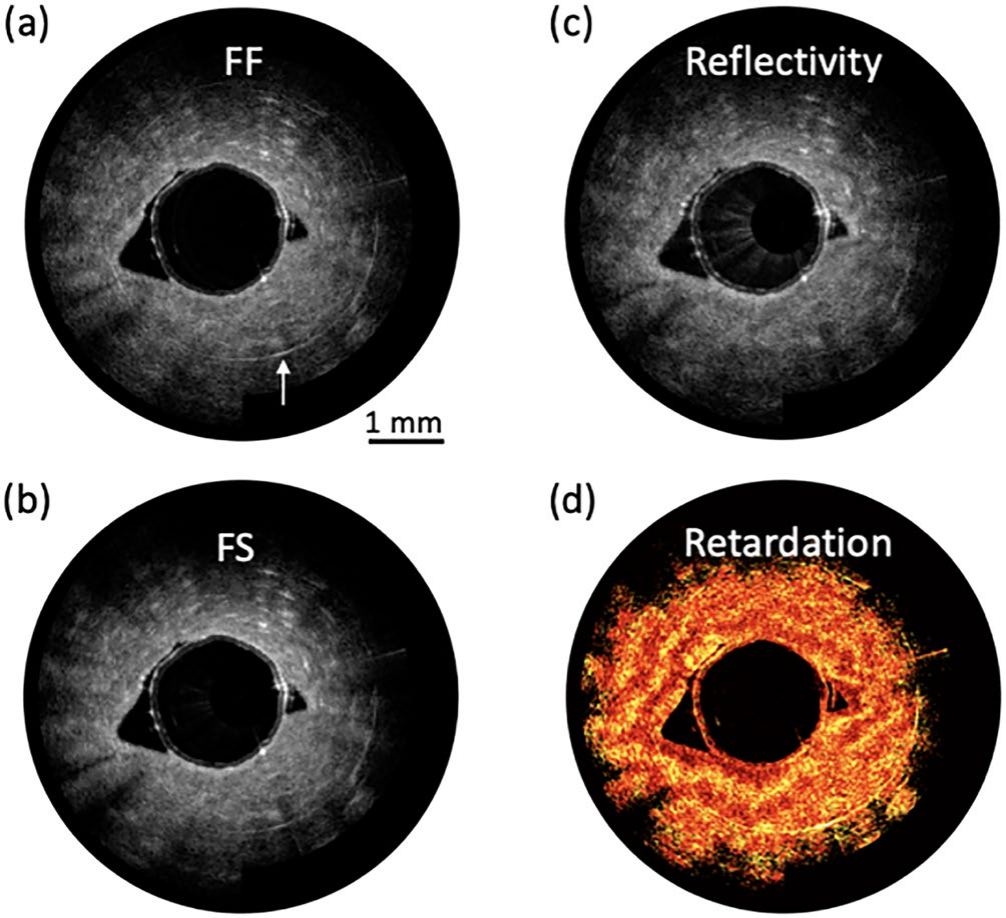
Images of chicken breast. (**a**) FF image, (**b**) FS image, (**c**) the normal image of reflectivity, and (**d**) the map of sample-field retardation of chicken breast. Scale bar: 1 mm. The image is generated by using Matlab. The figure is generated by using Matlab R2021a (Version: 9.10.0.1602886, https://www.mathworks.com/products/new_products/release2021a.html).

After the feasibility test, intravascular imaging probe (Fig. 5c) was connected to the PSOCT system to replace microscopic scanner (Fig. 2b) for further verification. First, a sliced chicken breast was fold into a cylinder for intravascular imaging. The imaging result is shown in Fig. 2. Figure 2a–d are FF image, FS image, reflectivity, and sample-field retardation of chicken breast, respectively. The effect of the sample birefringence is also clearly visualized in Fig. 2d.

**Figure 3:**
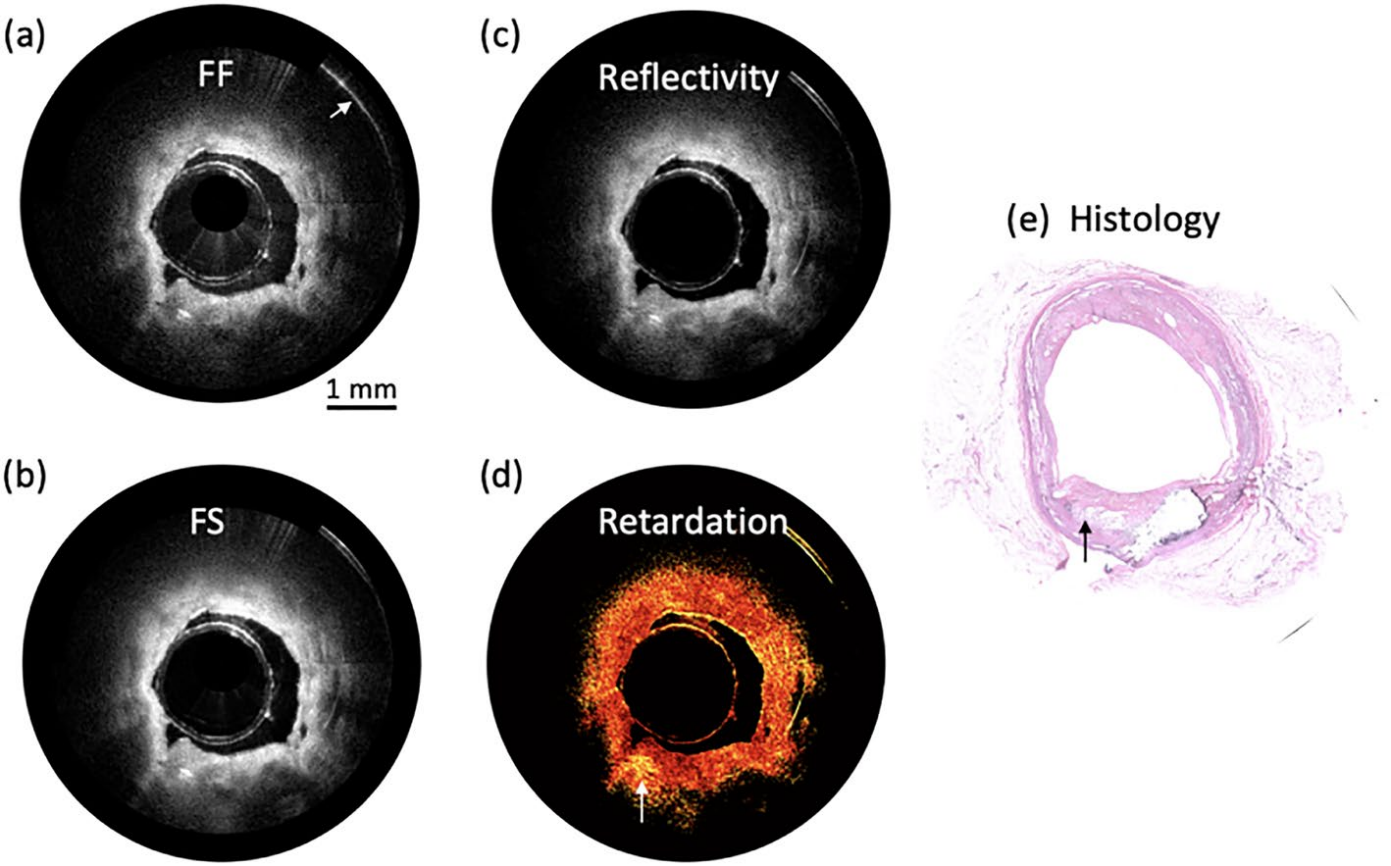
Images of atherosclerotic plaque. (**a**) FF image, (**b**) FS image, (**c**) the normal image of reflectivity, (**d**) the map of sample-field retardation, and (**e**) histology photo. Scale bar: 1 mm. Figures (**a**)–(**d**) are generated by using Matlab R2021a (Version: 9.10.0.1602886, https://www.mathworks.com/products/new_products/release2021a.html).

Then an atherosclerotic plaque from cadaver was imaged, as shown in Fig. 3. Fresh human coronary artery samples were obtained from cadavers and frozen in a − 19 degree freezer. After imaging, the tissue was decalcified, embedded, and sectioned to 6 μm-thick slides. Then the slides were stained with H&E.

Figure 3a–d are FF image, FS image, reflectivity, sample-field retardation, and histology of the plaque, respectively. In Fig. 3d, birefringence effect indicated by white arrow can be found, which corresponds to cholesterol crystals, as shown in Fig. 3e.

## Discussion

In this study we demonstrated a simple method to construct IV-PSOCT system. This was accomplished by introducing a long section of PMF, where two polarization modes can be separated spatially due to their different propagation speeds. Chicken tendon, chicken breast, and atherosclerotic plaque were imaged for the validation of IV-PSOCT. The imaging results demonstrated clear birefringence effect and different level birefringence.

 For clinical translation, there are a few of design improvements that can be implemented. First, the swept light source has a k-clock signal at ~ 400 MHz, allowing for an imaging range ~ 11 mm in standard OCT, or ~ 3.7 mm for each image [FF image, FS image, and slow–slow (SS) image in one Frame] when utilizing the proposed method. This imaging range may not be enough in large artery, such as aorta. In addition, an image overlap indicated by white arrow in Fig. 3a can be found. To address this issue, the imaging range can be increased through doubling the k-clock frequency or through dual edge sampling. In addition, a swept-source laser with a longer center wavelength, such as 1.7 μm, can be utilized to further improve penetration depth ^39–41^.

In our experiment, we used an extruded plastic tube for the sheath. Because of the fabrication process, it was birefringent similar to most of plastic tubes. In Fig. 3d, one can find the sheath image (inner circle) exhibits different degrees of retardation, which were caused by the inhomogeneous residual stress in the plastic tube. Because of its origin, the orientation of the birefringence is presumed to be parallel to the axis. It affected the obtained retardation in our PSOCT images, but it did not hinder our observation of local birefringence. For further improvement, a birefringent-free sheath will be investigated, or a calibration procedure will be introduced.

For probe design, current intravascular probe size is ~ 1.6 mm which is limited by the size of QWP (1 mm × 1 mm), hereby inhibiting in vivo intravascular imaging. This issue can be resolved with refined fabrication process. In addition, due to replicated OCT images, the artifacts (indicated by white arrow in Fig. 2a) caused by interfaces in a probe will also be replicated, which degraded OCT image quality. Therefore, a fused connection between PMF and GRIN lens will be applied to further reduce image artifacts.

In our study, a limited number of cadaver tissue were imaged for feasibility test. In the future, we will perform more ex/in vivo imaging of tissues with atherosclerotic plaques from cadavers and animal model and correlate the results with histological analysis. Furthermore, more quantitative analysis, such as decomposition and collagen density, will be investigated to provide a comprehensive evaluation of plaque. Finally, translation of this technology for in vivo application requires further miniaturization of probe.

In summary, we have reported IV-PSOCT system which is achieved by added a long section of PMF. The feasibility and the performance of our IV-PSOCT have been tested and validated using chicken tendon, chicken breast, and atherosclerotic plaque. The clear birefringence effect was visualized. With further improvement, we believe that proposed IV-PSOCT has great potential in intravascular imaging to pave a new path to evaluate the biomechanical properties of plaque. This will enhance the clinicians’ ability to identify vulnerable lesions, tailor interventional therapy, and monitor disease progression.

## Methods

### Principle

**Figure 4:**
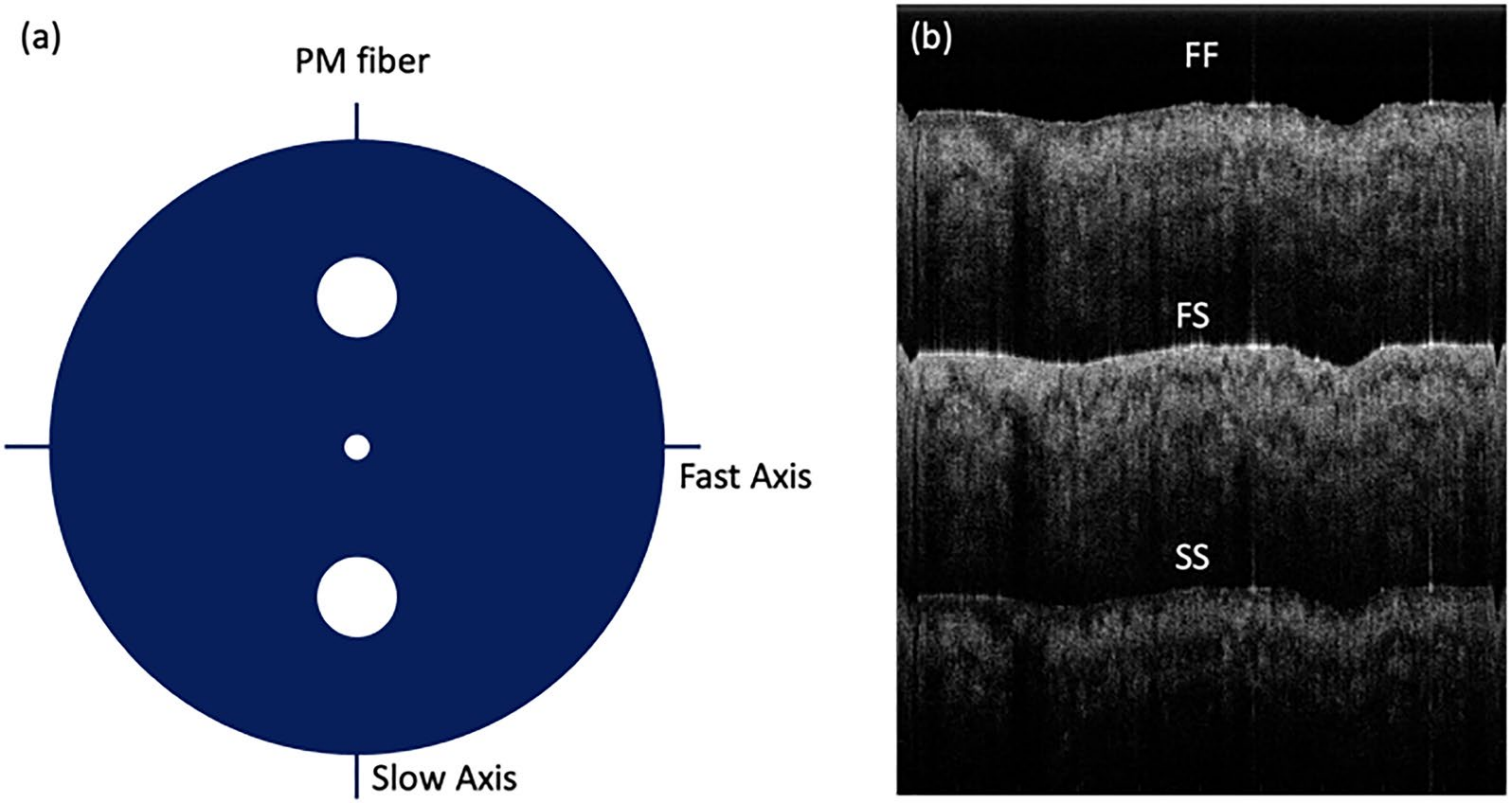
Definition of PMF axes (**a**) and FF, FS, and SS image replicas obtained by our IV-PSOCT system (**b**). Figure (**a**) is generated by using PowerPoint (Version 16.58, https://www.microsoft.com/en-us/microsoft-365/powerpoint). Figure (**b**) generated by using Matlab R2021a (Version: 9.10.0.1602886, https://www.mathworks.com/products/new_products/release2021a.html).

The PMF utilized in our system is highly birefringent with two linearly polarized (LP) eigenmodes. As shown in Fig. 4a, the light along the fast axis propagates faster than the light along the slow axis in the PMF. Therefore, three repeated OCT images can be generated, as shown in Fig. 4b. The FF image is a result of the OCT signal delivered through fast axis in the roundtrip. The SS image is produced through slow axis in the roundtrip. And the FS image is generated from the OCT signal delivered through fast axis in one way and slow axis in the other way. The axial separation Δz between the FF, FS, and SS images is determined by group delay of PMF. In our study, Δz was measured to be 2.7 mm. To obtain polarization composition of the sample, it is required to have the input state either LP*x* (fast axis) or LP*y* (slow axis). In our study, we applied a LPx input, in which FF (*I*_1_) and FS (*I*_2_) OCT images will be used. The OCT image of intensity can be reconstructed by $I_{OCT} = \sqrt {I_{1}^{2} + I_{2}^{2} }$, while the phase retardation map can be obtained by *I*_*PR*_ = arctan(*I*_1_/*I*_2_)^38^.

### System setup

**Figure 5:**
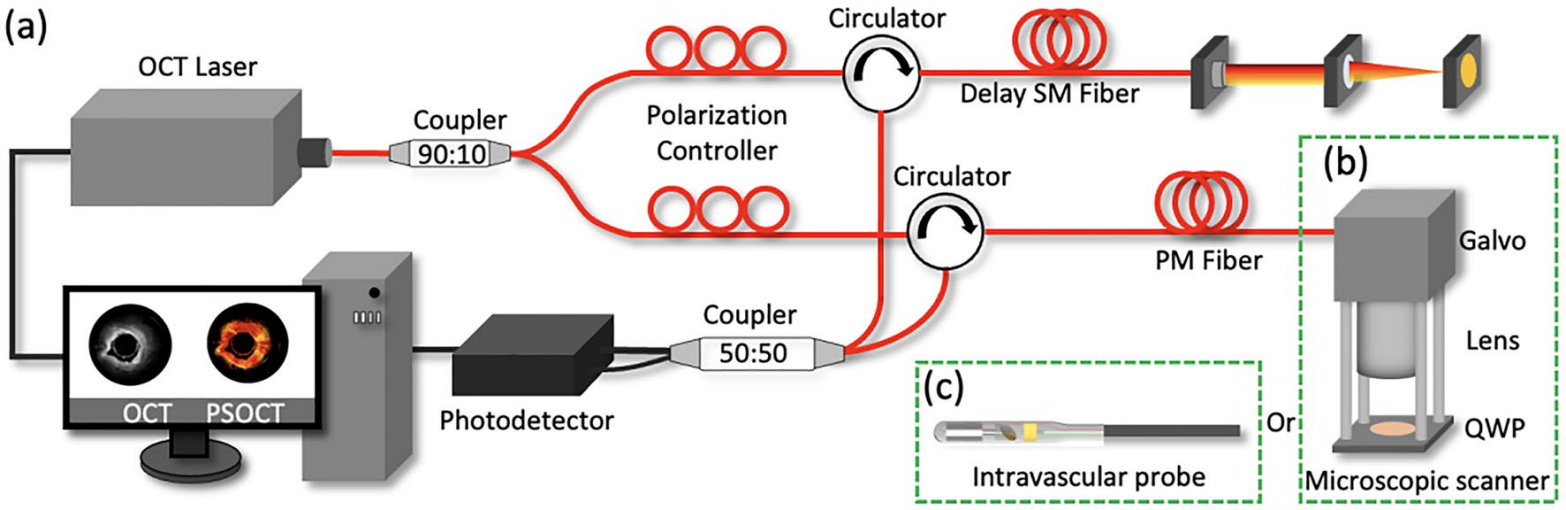
The schematic of IV-PSOCT system: (**a**) system setup, (**b**) scan head, and (**c**) intravascular imaging probe. *PM* polarization maintaining, *SM* single mode, *QWP* quarter-wave plate. The figure is generated by using PowerPoint (Version 16.58, https://www.microsoft.com/en-us/microsoft-365/powerpoint).

The schematic diagram of IV-PSOCT system is shown in Fig. 5. To incorporate PSOCT with the minimalistic system configuration, an ordinary OCT system based on a swept source was modified by introducing a 12-m long PMF in the sample arm^38^. This long section of fiber delay can temporally separate the signals in the two polarization states in the returning paths due to the difference in group delay. As well, a 12-m single mode optical fiber was added into the reference arm. The two temporally separated polarization states then interfered with the signal from the reference arm, resulting in two sets of intensity images, *I*_1_ (FF) and *I*_2_ (FS). We prepared two objective parts of a microscopic scanner (Fig. 5b) and an intravascular probe (Fig. 5c) for our system. An IV-PSOCT is constructed by equipping the intravascular probe at the distal end of the sample arm. The lateral resolution of microscopic and intravascular PSOCT is ~ 35 µm and ~ 30 µm, respectively. The axial resolution is ~ 9 µm. Both the clock signal and trigger signal output from the swept-source laser are also delayed by a 30-m coaxial cable to compensate the delay caused by the 12-m optical fibers. In addition, the wavelength dispersion was corrected using data postprocess^42^.

**Figure 6:**
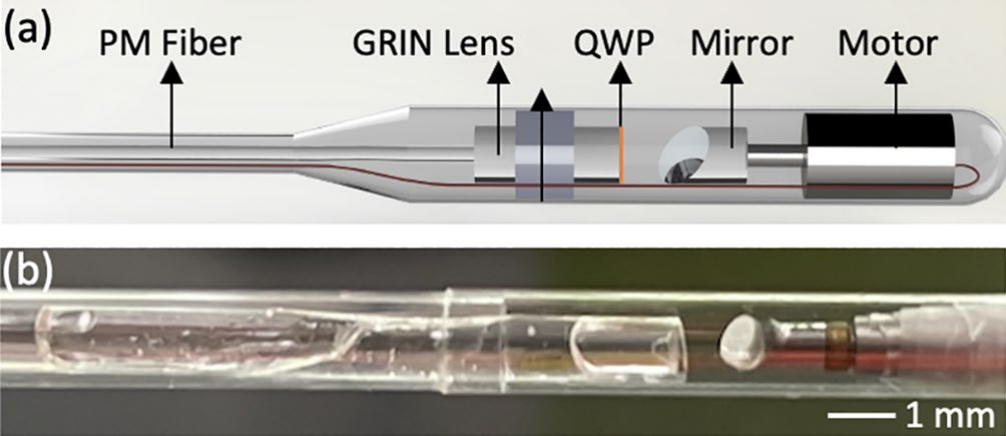
The schematic (**a**) and photo (**b**) of IV-PSOCT imaging probe. *PM* polarization maintaining, *GRIN* gradient index, *QWP* quarter-wave plate. Scale bar: 1 mm. Figure (**a**) is generated by using Solidworks (2020 SP3.0, https://blogs.solidworks.com/tech/2020/08/changes-in-solidworks-2020-sp3.html).

The schematic of our endoscopic probe is illustrated in Fig. 6. A PMF was used to deliver the OCT light to the probe. The full length of the PMF used in sample arm is 12 m. A gradient-index (GRIN) lens was equipped for focusing the OCT light at the sample for high resolution imaging. For the polarization state conversion, a custom-made quarter-wave plate (QWP) was attached to the GRIN lens. The orientation of the QWP was declined by 45° with respect to the fast axis of the PMF. The detailed fabrication process was described in Fig.8. The illumination light was then reflected by a mirror attached onto the shaft of a micromotor. The micromotor rotates the mirror for circumferential beam scanning. The micromotor-based beam scanning is advantageous in terms of imaging speed and imaging quality (non-uniform rotation distortion free) compared to the method based on a fiber rotary joint^43–45^.

### Pre-operational setup procedure for microscopic PSOCT

In the setup process before the operation of our system, the polarization controller in the sample arm was adjusted so that the illumination light is linearly polarized along the fast axis of the PMF at the distal end of the PMF. The quarter-wave plate then converts the linear polarization state into a circular polarization state, producing an equal distribution of the two linear polarization states at the sample. In the reference arm, the polarization controller was used for equal intensities of interference with the two polarization detection channels of *I*_1_ and *I*_2_.

The detailed procedure was described as below. A mirror was used as a target since it exhibits no polarization dependence in its reflectance.

**Figure 7:**
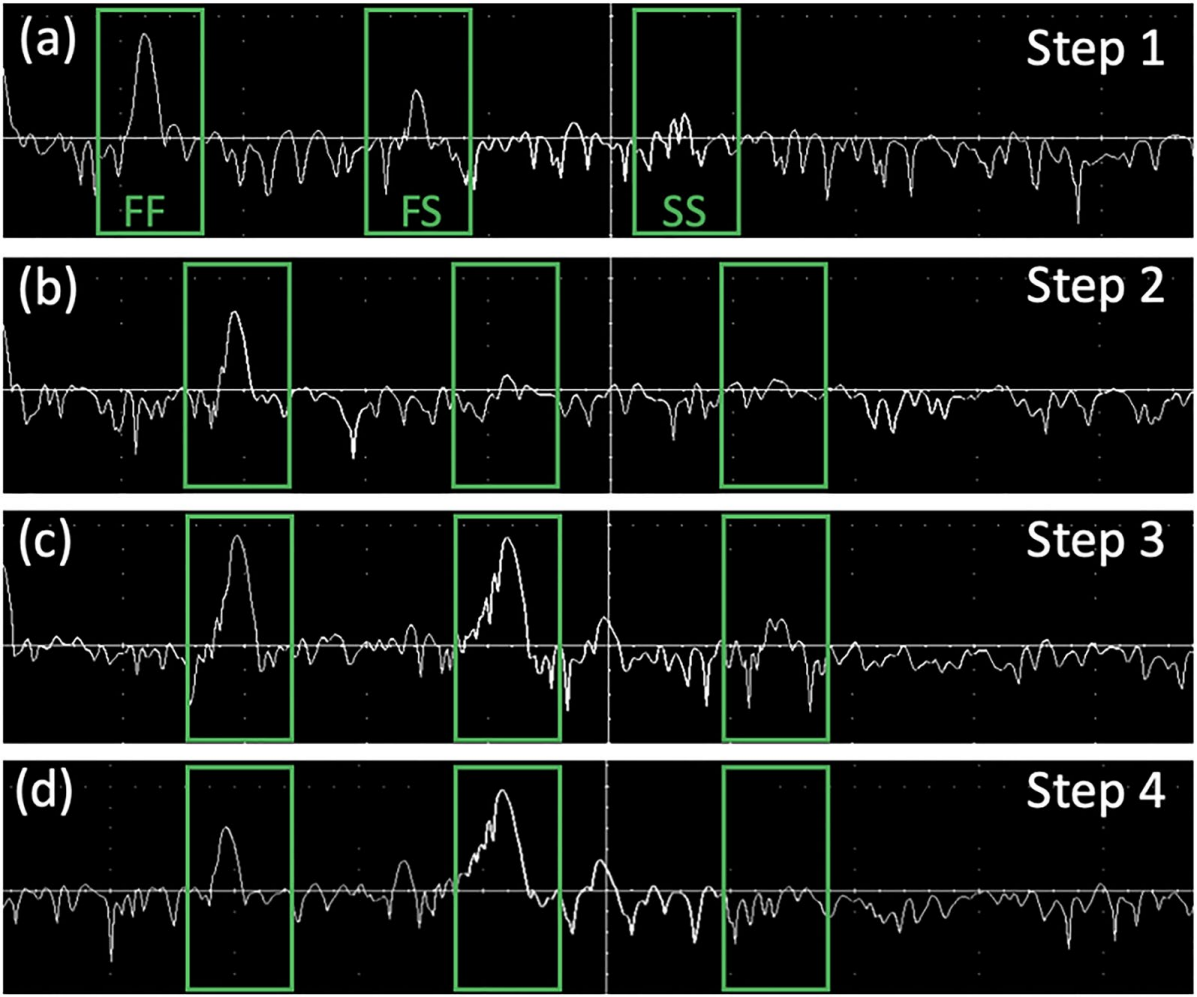
A-line signal from the pre-operational setup procedure of microscopic PSOCT at each step. (**a**) Step 1. (**b**) Step 2. (**c**) Step 3. (**d**) Step 4.

- Step 1: Adjust the polarization controllers in sample arm to minimize the amplitude of the SS image. Then, the input and the output of the PMF are completely along fast axis. The FF image becomes the brightest, as shown in Fig. 7a.
- Step 2: Insert a QWP between object lens and mirror. Set the angle of the QWP to be 0° with respect to the fast axis. QWP maximizes the amplitude of the FF image and minimizes that of the FS image, as shown in Fig. 7b.
- Step 3: Set the angle of the QWP to be 22.5° with respect to the fast axis. Then, the reflected is equally distributed to the fast axis and slow axis. Then adjust the polarization controllers in reference arm to equalize the amplitudes of the FF image and the FS image, as shown in Fig. 7c.
- Step 4—Set the angle of the QWP to be 45° with respect to the fast axis. Then, the sample-incident light is circularly polarized. The sample field recoupled to the PMF is completely along slow axis. The FF image becomes the darkest, while the FS image becomes the brightest, as shown in Fig. 7d.

### Alignment of QWP and pre-operational setup procedure for IV-PSOCT

In probe fabrication, the optics axis of QWP must be set as 45° with respect to the fast axis of PMF to obtain circularly polarized output light. The following steps are used to find the fast axis of PMF.

**Figure 8:**
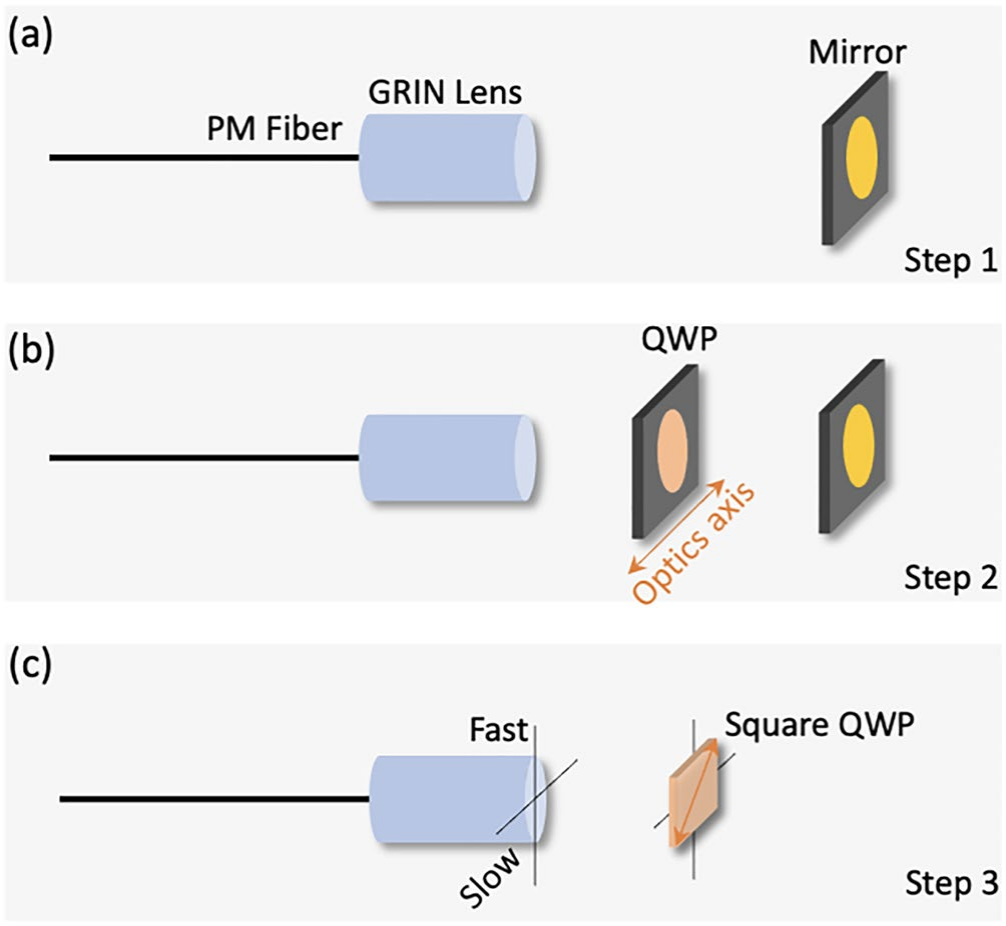
Detailed procedure of fabricating imaging probe. (**a**) Step 1. (**b**) Step 2. (**c**) Step 3. The figure is generated by using PowerPoint (Version 16.58, https://www.microsoft.com/en-us/microsoft-365/powerpoint).

- Step 1: Assemble PMF and GRIN lens first, and then place it in a rotation stage. A mirror was used as a target. Adjust the polarization controllers in sample arm to minimize the amplitude of the SS image. Then, the input and the output of the PMF are completely along fast axis. The FF image becomes the brightest (Fig. 8a).
- Step 2: Insert a QWP between GRIN lens and mirror. Adjust the angle of QWP to maximize the amplitude of the FF image and minimize that of the FS image. Then label the orientation of the optic axis of QWP as fast axis of PMF (Fig. 8b).
- Step 3: Assemble square QWP with GRIN lens. The optics axis of square QWP was set to be 45° with respect to the fast axis of PMF (Fig. 8c).

For IV-PSOCT pre-operational setup, we fabricated an additional probe which consist of only PMF and GRIN lens. The fast axis orientation of this probe and the one we performed experiment are same, which is achieved through a PMF mating sleeve.

- Step 1: Connect pre-operational probe with IV-PSOCT system and image a mirror. Adjust the polarization controllers in sample arm to minimize the amplitude of the SS image. Then, the input and the output of the PM fiber are completely along fast axis.
- Step 2: Connect intravascular probe with IV-PSOCT system and image a non-birefringence scattering sample. Adjust polarization controllers in sample arm for equalizing the amplitudes of the FF image and the FS image.

### Ethics approval

All methods were carried out in accordance with the University of California, Irvine (UCI) Institutional Review Board (IRB) and the Institutional Biosafety Committee (IBC). All experimental protocols were approved by the UCI IBC under protocol # 2009-1309, where informed consent was deemed unnecessary because confidentiality of the deceased cadaver tissues is protected and coded.

## Data Availability

The data that support the findings of this study are available from the corresponding author upon reasonable request.
